# Infant Perception of Incongruent Shapes in Cast Shadows

**DOI:** 10.1068/i0681

**Published:** 2015-04-01

**Authors:** Kazuki Sato, So Kanazawa, Masami K. Yamaguchi

**Affiliations:** Department of Psychology, Chuo University, Tokyo, Japan; and Japan Society for the Promotion of Science; Department of Psychology, Japan Women's University, Kanagawa, Japan; Department of Psychology, Chuo University, Tokyo, Japan

**Keywords:** cast shadow, familiarization-novelty procedure, infant, object perception, perceptual development

## Abstract

A cast shadow occurs when an object blocks the light from an illumination and projects a dark region onto a surface. Previous studies have reported that adults are slower to identify an object when the object has an incongruent cast shadow than when it has a congruent cast shadow (Castiello, 2001). Here, we used the familiarization-novelty preference procedure to examine whether 5- to 8-month-old infants could detect the relationship between object shapes and cast shadows. In Experiment 1, we examined the infants' ability to detect incongruency between objects and cast shadows. Results showed that 7- to 8-month olds could detect incongruence between the object shapes and the cast shadows, whereas 5- to 6-month olds did not. Yet, our control experiment showed that infants could not detect this incongruence from stimuli in which a white outline had been added to the original cast shadow to decrease the possibility of it being perceived as a cast shadow (Experiment 2). The results of these experiments demonstrate that 7- to 8-month olds responded to the congruence of cast shadows and to consistent contrast polarity between the cast shadow and its background.

## 1 Introduction

A cast shadow occurs when an object blocks the light from an illumination and projects a dark region onto a surface. A cast shadow is an effective cue for perceiving the three-dimensional (3D) information of objects such as distance, motion (Kersten, Mamassian, & Knill, 1997; Mamassian, Knill, & Kersten, 1998), and shape (Cavanagh & Leclerc, 1989; Norman, Dawson, & Raines, 2000).

Adult perception of cast shadows has been frequently studied. In Castiello's study, adult participants were asked to identify objects (Castiello, 2001). They found that the vocal reaction times were longer when the lighting, which is indicated by the attached shadows, and the shapes of the cast shadows were incongruent than when both were congruent. Additionally, adults were slower to identify objects when the objects were presented without cast shadows. These results suggest that the presence of attached and cast shadows affects object recognition in adults. Recently, Becchio, Mari, and Castiello (2010) extended the findings of Castiello (2001) by demonstrating that interference from an incongruent cast shadow also delayed object identification in typically developing 12-year olds. Comparing typically developing children and children with ASD, they showed that typically developing children showed the same response to an incongruent shadow as adults, while children with ASD needed more reaction time when the cast shadow was presented regardless of the congruency or incongruency of the cast shadow. In their study, typically developing children aged 12 years showed the ability to integrate cast shadows and the objects which cast them. Even for the typically developing population, the early emergence of this ability has not been examined. In the current study, we investigated whether they are able to integrate the cast shadows and the objects which cast them in infancy. In addition, some studies showed that adults could not perceive the cast shadow of a figure which had some property of its cast shadow changed. In a famous study, Hering's demonstration (Hering, 1874, 1964) suggested that putting a black line on the outline of a cast shadow to remove the penumbra, which is the fuzzy edge of a cast shadow, decreased the appearance of the cast shadow, making the cast shadow no longer perceptible. More recent studies have shown some conditions which decrease the appearance of a cast shadow, for example, putting a black or white outline on the penumbra, coloring the region of the cast shadow to white, or adding a texture different from the background on the region of the cast shadow (Hering, 1874, 1964; Kennedy & Bai, 2000; Rensink & Cavanagh, 2004).

To our knowledge, there have been only a few studies of infant perception of cast shadows (Imura et al., 2006; Van de Walle, Woodward, & Phillips, 1998; Yonas & Granrud, 2006). Van de Wall et al. (1998) investigated whether 5- and 8-month-old infants could perceive the relation between an object and its cast shadow. They examined whether infants could notice unnaturalness from a situation in which a cast shadow was moving separately from an object in a manner that adult participants had previously rated as unnatural shadow motion. Results indicated that infants showed preference for the unnatural movie in which an object and its shadow were moving separately. In this main experiment, the natural situation was static, but the unnatural situation was moving. This difference of stimuli should determine the infant's preference. In that case, the infants' preference was based on a motion rather than the unnaturalness of the shadow. They conducted two control experiments. In one control experiment, both the natural and the unnatural test stimuli were moving. The results revealed that infants preferred the natural stimuli, unlike the main experiment. This means that infants' preference was caused by “the principle of contact”; namely, the principle that objects should move together when they are in contact. As a result, Van de Walle and her coworkers demonstrated that infants could perceive the motion of shadows in accordance with the principle of contact rather than their ability for cast shadow perception.

Moreover, a study using a preferential reaching task (Yonas & Granrud, 2006) and a study using the habituate–dishabituate procedure to measure looking time (Imura et al., 2006) suggested that infants around 7 months of age can perceive depth from cast shadows. In Yonas & Granrud (2006), infants were presented with two objects with different cast shadows to test whether the infants could perceive the spatial layout from the cast shadows. One cast shadow was shifted away from the original position so that one object of that cast shadow was perceived as being nearer than the other. The results showed that 7-month-old infants preferred to reach for the nearer object. Imura et al. (2006) used the “ball-in-a-box” illusion (Kersten et al., 1997) to test whether infants can perceive the motion trajectory of objects from moving cast shadows. The “ball-in-a-box” animations were perceived as two different types of perceptive events depending on two different motions of the cast shadows: one gave the impression that the ball was receding in depth, while the other gave the impression that the ball was floating above the floor. They showed that 6- to 7-month-old infants could discriminate between these two types of illusory motion trajectories from the two different motions of the cast shadows.

Previous studies have consistently shown that infants around 7 months of age are able to perceive the spatial layout of a surface from cast shadows. However, how infants perceive the properties of a cast shadow itself remained unclear. Many studies showed that sensitivity for pictorial depth cues had already developed around 3- to 8 months (e.g., Bhatt & Bertin, 2001; Bhatt & Waters, 1998; Shuwairi, Albert & Johnson, 2007). Kavšek, Yonas, & Granrud (2012) reviewed these studies. In their review, 3- to 5-month-old infants had sensitivity to the pictorial depth cues, while infants over 6-months-old could perceive a 3D shape from these cues. They indicated that the ability to derive spatial information from pictorial depth cues had developed in 3- to 5-month olds, but that these infants could not yet use the depth cues to represent a 3D structure. Additionally, Slater, Mattock, & Brown (1990) found that even newborn infants could use the pictorial depth cues to perceive size constancy. In sum, as far as we know, infants around 5-month-old can perceive the distal spatial information by using pictorial depth cues and infants under 6-month-old cannot perceive a 3D representation from these cues.

In this study, we examined whether infants could perceive the constituents of cast shadows. We used stimuli which consisted of an object and its cast shadow. The shape of the cast shadow was either congruent or incongruent for each object. We tested whether infants could detect incongruence between the shape of the object and the shape of the cast shadow. We chose 5- to 8-month-old infants as the target age because previous studies showed that the perception of depth from cast shadows improves between 5 and 7 months of age (Imura et al., 2006; Yonas & Granrud, 2006). We used figures in which the shape of the object and the shape of the cast shadow were either congruent or incongruent. In two experiments, we examined infants' discrimination between the congruent and incongruent displays. We hypothesized that infants could detect the incongruence between the object and the cast shadow only if they could perceive the cast shadow as a shadow. To test this possibility, we used both cast shadow images and “non” cast shadow images by adding a white outline to the original cast shadow. In Experiment 1, we used 3D graphic cast shadow images as stimuli. In Experiment 2, we added a white outline to the cast shadow of the stimuli of Experiment 1 (noncast shadow images). Given previous findings with adults (Hering, 1874, 1964), we postulated that the image manipulation applied to Experiment 2 would make it difficult for infants to perceive the cast shadows as shadows. We hypothesized that when the infants could not perceive the cast shadow with the white outline as a cast shadow, they would be unable to detect the incongruence between the object and its cast shadow.

## 2 Experiment 1

In Experiment 1, we tested whether infants could detect the incongruent shape of a cast shadow. We created two kinds of stimuli: congruent figures and incongruent figures. The object in the congruent figures had its own cast shadow. In contrast, in the incongruent figures, the cast shadow of the object was replaced with a different cast shadow from another object. Infants were familiarized with two different images of the congruent figures (bottle and goblet). In the test phase, we presented the congruent figure and the incongruent figure. We hypothesized that if infants could detect the incongruent shape of a cast shadow, they would show novelty preference for the incongruent figure after the familiarization phase.

## 3 Method

### 3.1 Participants

Participants consisted of sixteen 5- to 6-month-old infants (8 males, mean age 169.2 days, ranging from 149 to 192 days), and sixteen 7- to 8-month-old infants (8 males, mean age 230.3 days, ranging from 199 to 253 days).

Additional infants were tested in this experiment, but were excluded from analysis due to fussiness (2), failure to look at the monitor for less than 60% of the familiarization phase (3), a side bias greater than 90% (2), or failure to maintain eye contact with the camera due to standing (1).

### 3.2 Apparatus

Throughout the experiment, all stimuli were displayed on a 21-inch color CRT monitor. The infant and the CRT monitor were located inside an enclosure made of iron poles and covered with cloth. The distance between the infants and the monitor was approximately 40 cm. A loud speaker was positioned on both sides of the CRT monitor. There was a pinhole CCD camera just below the monitor screen. Throughout the experiment, the infant's behavior was videotaped through this camera. The experimenter could observe the infant's behavior via a TV monitor connected to a pinhole camera.

### 3.3 Stimuli

We created two kinds of images, a bottle and a goblet, using the 3D rendering package POVRay (Persistence of Vision Raytracer). The bottle and the goblet were illuminated by an ambient and point light source from the upper left. Shadow images were generated by removing the objects rendering the image, leaving only the shadow. The stimuli images were created by digitally combining the shadows and the object images of the bottle and the goblet using Adobe Photoshop. The color of the bottle and color of the goblet were matched in chromaticity (*x* = 0.407, *y* = 0.431). The reflectance model used a specular reflectance of 0.25 and a diffuse reflection of 0.70.

**Figure 1.**
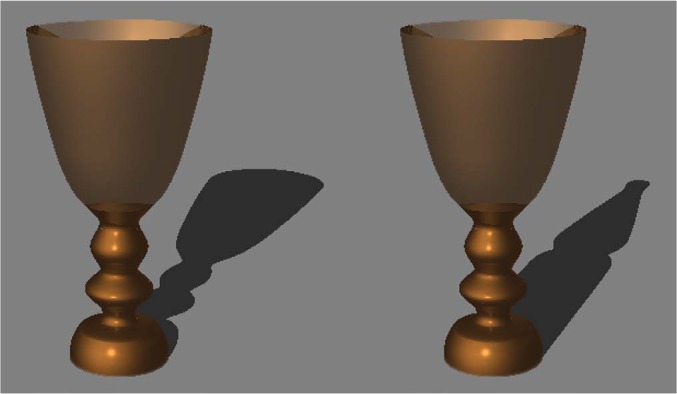
Example of the congruent figure (left) and the incongruent figure (right) used in Experiment 1. In the incongruent figure, the object has the cast shadow of another object.

We used the bottle and goblet images as the congruent figures. In order to create the incongruent figures, we replaced the goblet's shadow with the bottle's shadow (Figure 1). The width and height of the congruent figures were 10.29° and 16.64° for the bottle and 14.95° and 16.78° for the goblet. The width and height of the incongruent figures were 12.55° and 16.64° for the bottle and 12.70° and 16.78° for the goblet. All stimuli were depicted on a midgray (18.50 cd/m^2^) background. The mean luminance within the object region and the cast shadow region were 8.66 cd/m^2^ for the bottle, 3.30 cd/m^2^ for the goblet, 0.55 cd/m^2^ for the cast shadow of the bottle, and 0.57 cd/m^2^ for the cast shadow of the goblet.

### 3.4 Procedure

**Figure 2.**
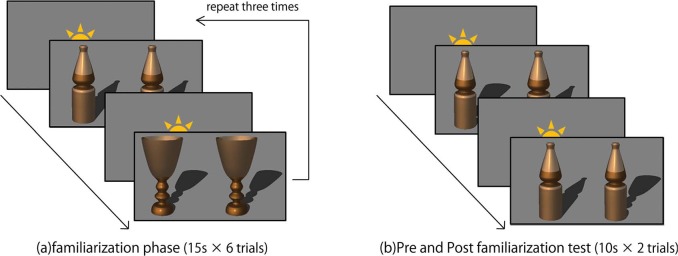
(a) In familiarization phase, we presented congruent figures. In each trial, we used goblet or bottle by turns. The order of the stimuli was counterbalanced across infants. (b) In pre- and post-familiarization test, we presented congruent and incongruent figures. The object types and the position of stimuli were counterbalanced across infants.

We used the familiarization-novelty preference procedure to test whether infants could detect incongruence between the objects and the cast shadows. The experiment consisted of three phases: the pre-familiarization test, the familiarization phase, and the post-familiarization test (Figure 2). Infants first participated in pre-familiarization test trials in order to check their spontaneous preference. After that, they participated in familiarization trials, followed immediately by post-familiarization test trials.

During the familiarization phase, we presented the congruent figures side by side for 15 s in six trials. Half of the infants of both age groups were familiarized with the congruent figure of the bottle in odd-numbered trials and with the congruent figure of the goblet in even-numbered trials. The other infants were familiarized with the goblet in odd-numbered trials and with the bottle in even-numbered trials. We presented two types of congruent figures during familiarization in order to facilitate infants' extraction of the computer-generated 3-dimensional object–shadow relationship rather than the local image properties.

We tested the infants' preference for the incongruent figure in the pre-familiarization and post-familiarization phases. In the pre- and post-test phases, the congruent figures and the incongruent figures were presented side by side for 10 s in two trials. The position and order of the stimuli were counterbalanced across infants. In each presentation, half of the infants of both age groups were tested with the bottle, and the other half were tested with the goblet.

Prior to a trial in each phase, a cartoon with a short sound was presented at the center of the monitor to get the infant's attention. The experimenter observed the infants' looking behavior by monitor and initiated the trial when the infant was looking at the cartoon.

### 3.5 Data coding

One observer, unaware of the stimulus identity, measured each infant's looking time for the right or left presentation field from recorded video movies. To calculate interobserver agreement, a second observer's measurement of the infants' looking time was obtained from the 31.25% of total data. Interobserver agreement was *r* = 0.922 across Experiment 1.

## 4 Results & Discussion

### 4.1 Familiarization trials

For the familiarization phase, individual looking times were averaged across the first three and the last three trials. In the first three trials, the mean total looking time of each group was 13.5 s for infants aged 5- to 6 months, and 12 s for infants aged 7- to 8 months. In the last three trials, the mean total looking time of each group was 11.24 s for infants aged 5- to 6 months, and 10.64 s for infants aged 7- to 8 months. The individual looking times were averaged across the first three and the last three trials.

To examine whether the age or the sex of the infant had affected the degree of familiarization, we performed a three-way ANOVA with trial (the first three, the last three) as a within-participants factor X age group (5- to 6 months, 7- to 8 months), as a between-participants factor X sex (male, female), and as a between-participants factor. The ANOVA revealed a significant main effect of the trial: *F*(1, 28) = 21.9, *p*<.001, η_p_^2^ = 0.44. This means a significant decrease in looking times over the trials. Significant main effect of the age was observed: *F*(1, 28) = 4.20, *p* = 0.049, η_p_^2^ = 0.13. This means that looking times were significantly longer in 5- to 6 months than 7- to 8 months. No other effects were reliable: all ns, *p* > .05. These results showed that participants in each age group were familiarized to the congruent figure and that there was no significant difference in the decrease in looking times between sexes.

### 4.2 Test trials

The mean total looking time of each group was 18.42 s for infants aged 5- to 6 months, and 16.71 s for infants aged 7- to 8 months in the pre-familiarization test. In the post-familiarization test, the mean total looking time of each group was 15.64 s for infants aged 5- to 6 months, and 14.88 s for infants aged 7- to 8 months.

**Figure 3.**
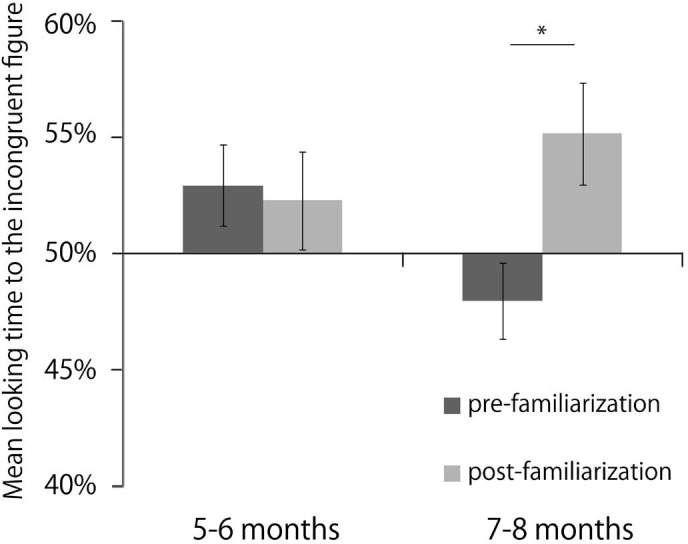
Mean percentage of looking time for the incongruent figure in the pre-familiarization test (black bars) and in the post-familiarization test (light gray bars) of Experiment 1. Error bars indicate standard errors. *p < 0.01. The data are collapsed across object types.

In the pre- and post-familiarization tests, we calculated the preference score for the incongruent figure based on the looking times of each infant. The preference score was the ratio of the looking times for the incongruent figures to the total looking times for both the congruent and incongruent figures. Figure 3 shows the mean preference score. As a preliminary analysis, a two-way ANOVA was performed with test (pre-familiarization test, post-familiarization test) and sex (male, female) as between-participants factor. This ANOVA showed no significant main effect (*p*> 0.05) or interaction (*p*> 0.05). Thus, we excluded the sex factor from the next analyses.

To examine the significance of the preference for the incongruent figure in each age group, a two-tailed *t*-test verses chance (50%) was performed. This analysis revealed that 7- to 8-month-old infants showed a significant preference for the incongruent figures in the post-familiarization test, *t*(15) = 2.33, *p* = 0.03, *r* = 0.52, but not in the pre-familiarization test, *t*(15) = 1.24, ns, *r* = 0.31, 5- to 6-month-old infants showed no significant preference in either the pre-familiarization test, *t*(15) = 1.70, ns, *r* = 0.40, or the post-familiarization test, *t*(15) = 1.09, ns, *r* = 0.27.

To examine whether infants looked longer at the incongruent figure in the post-familiarization test than in the pre-familiarization test, a three-way ANOVA was performed with test (pre-familiarization test, post-familiarization test) as a within-participants factor X age group (5- to 6 months, 7- to 8 months), as a between-participants factor X object type (goblet, bottle), and as a between-participants factor. The ANOVA showed that a significant main effect of test was observed: *F*(1, 28) = 5.04, *p* = 0.03, η_p_^2^ = 0.15. Significant interaction was observed [test X age group: *F*(1,28) = 6.11, p=0.02, η_p_^2^ = 0.18. No other effects were reliable: all ns, *p* > 0.05. To explore what drove the test X age group interaction, we performed a post-hoc analysis. The simple effects analyses showed that the preference scores of the 7- to 8-month olds were significantly different between the post-familiarization test and the pre-familiarization test: *F*(1, 14) = 15.73, *p* = 0.001, η_p_^2^ = 0.53. This result reflected that 7- to 8-month olds looked longer at the incongruent figure in the post-familiarization test than in the pre-familiarization test. This test X age group interaction was not different for the two different object types. This simple effects analyses also showed that the preference score of the pre-familiarization test differed by age group: *F*(1, 28) = 4.93, *p* = 0.03, η_p_^2^ = 0.15. However, all infants' preferences in pre-familiarization test were not significant from chance level.

We found that only the 7- to 8-month-old infants showed the novelty preference for incongruent figures after familiarization to the congruent figures. These results suggest that the 7- to 8-month-old infants could detect the incongruence between the shape of an object and its cast shadow, but the 5- to 6-month-old infants could not detect this incongruence.

## 5 Experiment 2

The results of Experiment 1 showed novelty preference in 7- to 8-month-old infants for the incongruent figures after they were familiarized with the congruent figures. This result suggests that the 7- to 8-month-old infants could perceive the cast shadows. In Experiment 2, we examined whether the infants' discrimination of congruency between the object and its cast shadow depended on their perception of the cast shadow as a shadow.

In Experiment 2, we added a white outline to the cast shadow of the stimuli used in Experiment 1. Previous studies have reported that adult observers could not perceive the cast shadow from such stimuli (Hering, 1874, 1964). We used the same familiarization-novelty procedure as in Experiment 1, checking infants' preference for congruent and incongruent figures after they were familiarized with the congruent figures.

## 6 Method

### 6.1 Participants

Participants consisted of sixteen 5- to 6-month-old infants (9 males, mean age 169.6 days, ranging from 137 to 193 days), and sixteen 7- to 8-month-old infants (9 males, mean age 229.9 days, ranging from 196 to 251 days).

An additional nine infants were tested in this experiment, but were excluded from analysis due to fussiness (3), failure to look at a monitor for less than 60% of the familiarization phase (4), low birth-weight (1), or a side bias greater than 90% (2).

### 6.2 Stimuli

**Figure 4.**
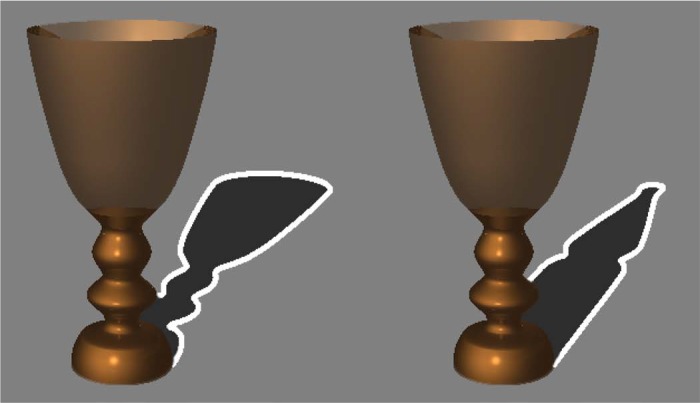
Example of the congruent figure with outline (left) and the incongruent figure with outline (right) used in Experiment 2. We added a white outline to the cast shadow of the stimuli used in Experiment 1.

To decrease the appearance of the shadows, we added a white outline to the cast shadow region of the stimuli of Experiment 1 (Figure 4). All outlines were colored white (luminance = 106.2 cd/m^2^) and the thickness of the lines were 0.3° of visual angle; 0.15° on the inside of the cast shadow and another 0.15° on the outside of the cast shadow. The mean luminance within the cast shadow region was 14.75 cd/m^2^ for the cast shadow of the bottle, and 15.58 cd/m^2^ for the cast shadow of the goblet. In both the familiarization and test trial, we used the stimuli which added the white outline to the cast shadow.

### 6.3 Apparatus, and procedure

The apparatus and procedure were the same as those in Experiment 1.

### 6.4 Data coding

To calculate interobserver agreement, a second observer's measurement of the infants' looking time was obtained from 31.25% of total data. Interobserver agreement was *r* = 0.892 across this experiment.

## 7 Results & Discussion

### 7.1 Familiarization trials

For the familiarization phase, individual looking times were averaged across the first three and the last three trials. In the first three trials, the mean total looking time of each group was 12.56 s for infants aged 5- to 6 months, and 12.76 s for infants aged 7- to 8 months. In the last three trials, the mean total looking time of each group was 10.51 s for infants aged 5- to 6 months, and 10.21 s for infants aged 7- to 8 months. The individual looking times were averaged across the first three and the last three trials.

As in Experiment 1, we performed a three-way ANOVA with trial (the first three, the last three) as a within-participants factor X age group (5- to 6 months, 7- to 8 months), as a between-participants factor X sex (male, female), and as a between-participants factor to examine any potential difference in the amount of familiarization between age, group, or sex. The ANOVA revealed a significant decrease in looking times over trials [a significant main effect of the trial: *F*(1, 28) = 47.75, *p* < 0.001, η_p_^2^ = 0.63.]. No other effects were reliable: all ns, *p* > 0.05. These results showed that participants in each age group and sex were familiarized to the congruent figure to a similar degree.

### 7.2 Test trials

**Figure 5.**
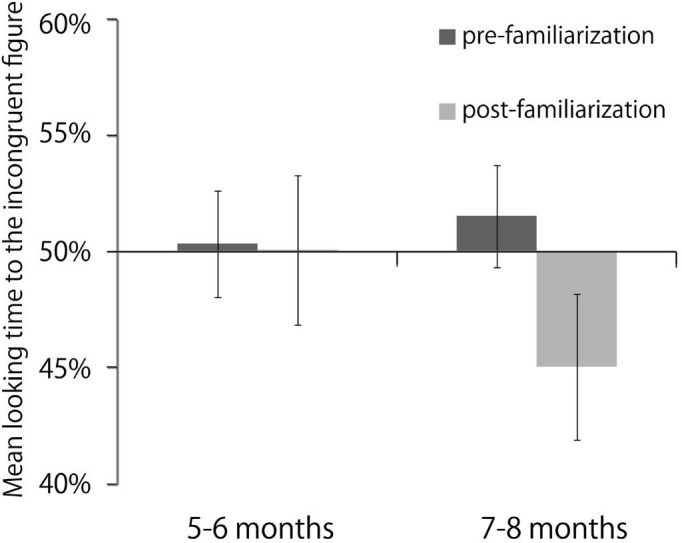
Mean percentage of looking time for the incongruent figure in the pre-familiarization test (black bars) and in the post-familiarization test (light gray bars) of Experiment 2. Error bars indicate standard errors. The data are collapsed across object types.

The mean total looking time of each group was 18.19 s for infants aged 5- to 6 months, and 17.61 s for infants aged 7- to 8 months in the pre-familiarization test. In the post-familiarization test, the mean total looking time of each group was 14.61 s for infants aged 5- to 6 months, and 13.67 s for infants aged 7- to 8 months. In the pre- and post-familiarization tests, we calculated the preference score for the incongruent figure based on the looking time of each infant. Figure 5 shows the mean preference scores. As a preliminary analysis, a two-way ANOVA was performed with test (pre-familiarization test, post-familiarization test) and sex (male, female) as between-participants factors. This ANOVA showed no significant main effect (*p* > 0.05) or interaction (*p* > 0.05). Thus, we excluded the sex factor from the next analyses.

To examine the significance of the preference for the incongruent figure in each age group, a two-tailed *t*-test verses chance (50%) was performed. This analysis revealed that preference for the incongruent figures were not shown in all ages (5- to 6-month olds in pretest: *t*(15) = 0.15 ns, *r* = 0.04; 5- to 6-month olds in post-familiarization test: *t*(15) = 0.02, ns, *r* = 0.01; 7- to 8-month olds in pre-familiarization test: *t*(15) = 0.69, ns, *r* = 0.18; 7- to 8-month olds in post-test: *t*(15) = 1.57, ns, *r* = 0.38).

A three-way ANOVA with test (pre-familiarization test, post-familiarization test) as a within-participants factor X age group (5- to 6 months, 7- to 8 months), as a between-participants factor X object type (goblet, bottle), and as a between-participants factor was performed. The ANOVA indicated that no effect was significant: all ns, *p* > 0.05 [main effect of test: *F*(1, 28) = 0.23, *p* = 0.63, η_p_^2^ = 0.01; main effect of age group: *F*(1, 28) = 1.14, *p* = 0.29, η_p_^2^ = 0.04; main effect of object type: *F*(1, 28) = 2.44, *p* = 0.13, η_p_^2^ = 0.08].

Unlike in Experiment 1, we found no significant difference between the preference score of the pre and post-familiarization tests in Experiment 2. The only difference between Experiments 1 and 2 was whether the cast shadows in the stimulus images were accompanied with a white outline. Previous study showed that adult observers could not perceive a cast shadow as a shadow when it was enclosed with a white outline (Hering, 1874, 1964). The successful discrimination of incongruence between the shape of an object and the shape of its cast shadow by 7- to 8-month-old infants in Experiment 1, but not in Experiment 2, suggests that such discrimination is possible for 7- to 8-month-old infants only when the cast shadow is perceived. In turn, this suggests that 7- to 8-month-old infants' detection of the incongruence between the shape of an object and the shape of its cast shadow in Experiment 1 was based on the perception of the cast shadow.

## 8 General discussion

In this study, we have revealed that infants can perceive cast shadows by testing whether infants could detect the incongruence between the shape of an object and a cast shadow. Infants were familiarized with the stimuli of congruent figures in which the object has its own cast shadow, and were then tested for novelty preference with congruent and incongruent figures. We hypothesized that if infants could detect the congruence of the object and its cast shadow, they would show novelty preference for the incongruent figure after the familiarization phase. In Experiment 1, 7- to 8-month-old infants showed novelty preference for the incongruent figures after being familiarized with congruent figures. However, when white outlines were added to the cast shadows (Experiment 2), the infants showed no novelty preference for the incongruent figure. The results of Experiments 1 and 2 demonstrate that the 7- to 8-month-old infants could detect the incongruent shape of a cast shadow only when the cast shadows could be perceived as a shadow.

The results of our experiments are consistent with previous studies on the development of the perception of cast shadows (Imura et al., 2006; Yonas & Granrud, 2006). Yonas and Granrud (2006) investigated whether 5-month-old infants and 7-month-old infants could use a cast shadow as a pictorial depth cue by measuring infants' reaching behavior under the monocular viewing condition and the binocular viewing condition. In their previous study, they found that pictorial depth cues were more effective in the monocular viewing condition than in the binocular viewing condition for eliciting the reaching behavior of infants (Granrud, Yonas, & Opland, 1985). Yonas and Granrud (2006) showed that 7-month-old infants preferentially reached for the apparently nearer images specified by a cast shadow cue, and that such preference was stronger under the monocular viewing condition than under the binocular viewing condition. In contrast, 5-month-old infants did not show any preference for either condition. Moreover, Imura et al. (2006) revealed that 6- to 7-month-old infants could discriminate the motion trajectories of an object from the motion of the cast shadow by using the “ball-in-a-box” animations (Kersten et al., 1997). Imura et al. (2006) showed that 6- to 7-month-old infants could discriminate between those two types of motion trajectories from the motion of the cast shadow, but that 4- to 5-month-old infants could not. These two studies suggest that the perception of the cast shadow emerges at least around 7 months of age. Consistent with these studies, our results suggest that the ability to perceive cast shadow and detect congruency between the shape of an object and its cast shadow develops by 7 months of age. In the future, a new approach or new measurement technique may elicit the perception of shadows from 5- to 6-month-old infants.
